# The Function of SARI in Modulating Epithelial-Mesenchymal Transition and Lung Adenocarcinoma Metastasis

**DOI:** 10.1371/journal.pone.0038046

**Published:** 2012-09-26

**Authors:** Changli Wang, Yanjun Su, Lianmin Zhang, Meng Wang, Jian You, Xiaoliang Zhao, Zhenfa Zhang, Jun Liu, Xishan Hao

**Affiliations:** Department of Lung Cancer, Tianjin Medical University of Cancer Institute and Hospital, Tianjin, China; Cincinnati Children's Hospital Medical Center, United States of America

## Abstract

The *SARI* (suppressor of AP-1, regulated by IFN) gene, which is also called *BATF2*, is associated with the risk of several kinds of cancer, and loss of SARI expression is frequently detected in aggressive and metastatic cancer. However, the functional role of SARI in lung adenocarcinoma remains unknown. We have shown that loss of SARI expression initiates epithelial-mesenchymal transition (EMT), which is visualized by repression of E-cadherin and up-regulation of vimentin in lung adenocarcinoma cell lines and in clinical lung adenocarcinoma specimens. Using a human lung xenograft-mouse model, we observed that knocking down endogenous SARI in human carcinoma cells leads to the development of multiple lymph node metastases. Moreover, we showed that SARI functions as a critical protein in regulating EMT by modulating the (GSK)-3β-β-catenin signaling pathway. These results demonstrate the mechanism of SARI function in EMT and suggest that assessment of SARI may serve as a prognostic biomarker and potential therapeutic target for lung adenocarcinoma metastasis.

## Introduction

Lung cancer is the leading cause of cancer death worldwide [1], and lung adenocarcinoma is the most common type of lung cancer [2]. In the absence of metastasis, lung adenocarcinoma is largely a treatable disease. Thus, the early diagnosis of patients who develop lung adenocarcinoma metastasis could reduce the mortality and morbidity associated with this disease. The development of metastasis depends on the migration and invasion of cancer cells from the primary tumor into the surrounding tissues. To acquire such invasive abilities, carcinoma cells may acquire unique phenotypic changes such as epithelial-mesenchymal transition (EMT). EMT is a highly conserved cellular process that allows polarized, generally immotile epithelial cells to convert to motile mesenchymal-appearing cells. This process was initially recognized during several critical stages of embryonic development and has more recently been implicated in promoting carcinoma invasion and metastasis [3]. During EMT, 3 major changes occur: (i) morphological changes from a cobblestone-like monolayer of epithelial cells to dispersed, spindle-shaped mesenchymal cells with migratory protrusions; (ii) changes in the expression of differentiation markers, including cell-cell junction proteins, cytokeratin intermediate filaments, vimentin filaments and fibronectin; and (iii) acquisition of invasiveness through the extracellular matrix [4], [5], [6], [7], [8].

Decreased E-cadherin expression or gain of vimentin expression is closely correlated with various indices of lung adenocarcinoma progression, including the grade, local invasiveness, dissemination into blood, and tumor relapse after radiotherapy [9], [10].

SARI, also known as suppressor of AP-1, is regulated by IFN and has been implicated in cell-growth inhibition and apoptosis. SARI is down-regulated in various types of human cancers and plays an important role in tumor development [11], [12].

Thus, it is very likely that SARI functions as a tumor suppressor in cancer development; however, its role and mechanism in lung adenocarcinoma metastasis is largely unknown. In the current study, we have shown that the loss of SARI facilitates EMT, leading to lung adenocarcinoma metastasis.

## Results

### SARI Regulates EMT In Vitro

**Figure 1:**
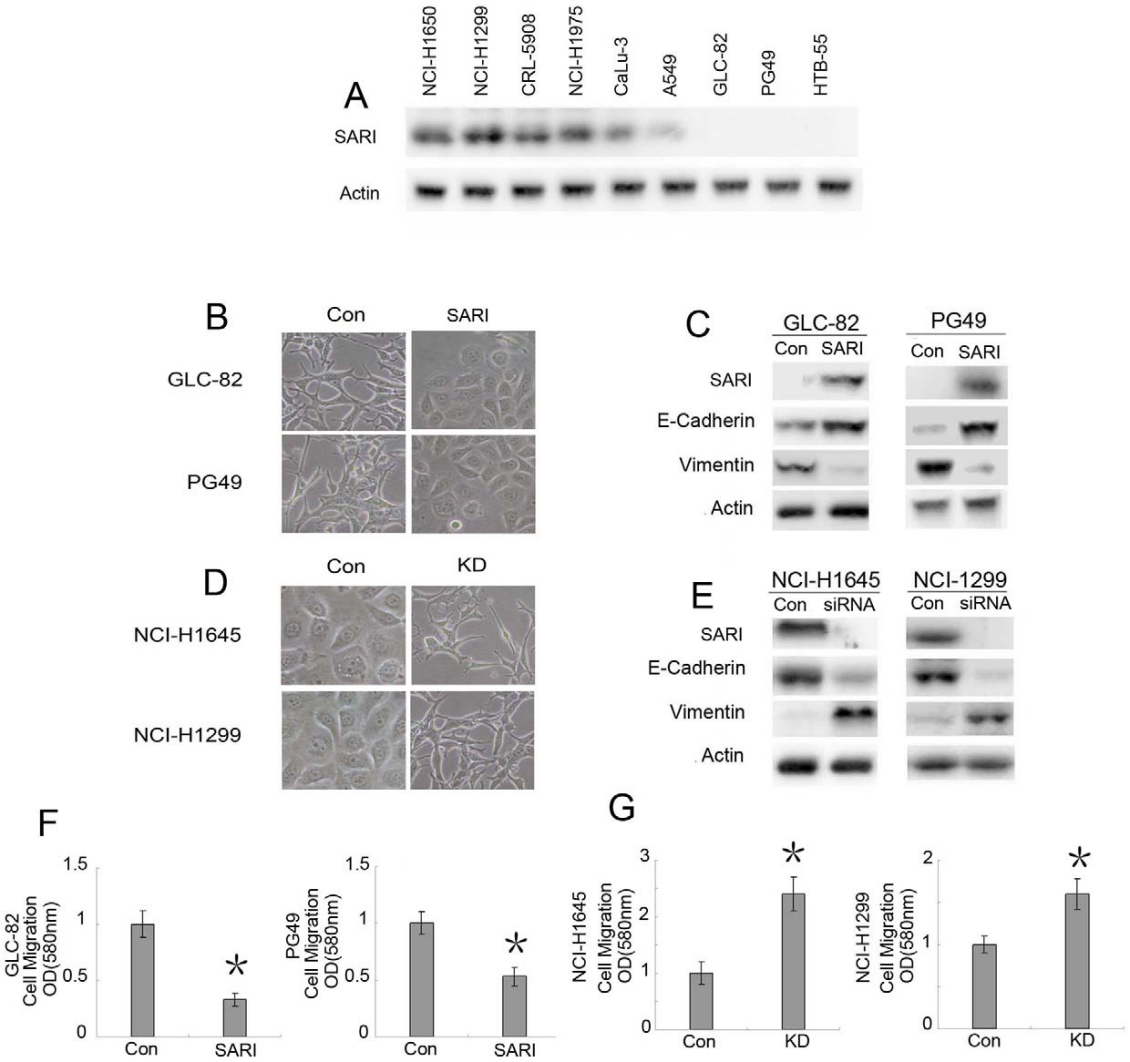
SARI regulates EMT in various cell lines. SARI expression was detected in lung adenocarcinoma cell lines: (A) NCI-H1650, NCI-H1299, and CRL-5908 cells express SARI very highly; NCI-H1975, CaLu-3, and A549 cells express lower levels of SARI; and GLC-82, PG49, and HTB-55 cells do not express SARI at all. Elevated SARI levels reverse EMT in vitro. (B) The morphology of GLC-82 and PG49 cells transfected with either the control vector or SARI was revealed by phase-contrast microscopy (magnification: 100×). (C) The expression of epithelial or mesenchymal markers in GLC-82 and PG49 cells transfected with either the control vector or SARI was analyzed by western blotting. β-Actin was used as a loading control. (D) Knockdown of SARI initiates EMT in vitro. NCI-H1650 and NCI-H1299 cells were infected with control lentivirus or lentivirus-expressing shRNA specific to SARI and then selected with puromycin. The morphology was revealed by phase-contrast microscopy (magnification: 100×). (F) Effect of SARI on cell migration in vitro. GLC-82 and PG49 cells transfected with either the control vector or SARI were plated in transwell chambers for 48 h, and quantitative measurements of migratory cells were determined. The data are presented as the mean ± SEM of each sample measured in triplicate (p<0.01). (G) Increased mesenchymal and reduced epithelial markers in SARI-knockdown cells were analyzed by western blotting (p<0.01).

We detected SARI expression in lung adenocarcinoma cell lines and found that NCI-H1650, NCI-H1299, and CRL-5908 cells express SARI very highly; NCI-H1975, CaLu-3, and A549 cells express less SARI; and GLC-82, PG49, and HTB-55 cells do not express SARI at all (Fig. 1A). In addition, we established whether SARI can regulate EMT. Cells undergoing an EMT or mesenchymal-epithelial transition (MET) experience transient morphological and biological changes that affect cell polarity, contact with neighboring cells, and cell motility [13], [14], [15], [16], [17]. These phenotypic changes are reminiscent of GLC-82 and PG49 cells, which when transfected with SARI display a clear morphological transition from spindle-like fibroblastic (control) to cobblestone-like cells (transfected with SARI) with well-organized cell contact and polarity (Fig. 1B). Increased E-cadherin and reduced vimentin expression due to SARI expression was observed in GLC-82 and PG49 cells (Fig. 1C). In contrast, when endogenous SARI expression in 2 different human lung adenocarcinoma cell lines (NCI-H1650 and NCI-H1299) was knocked out, EMT was clearly detected based on changes in cell morphology and biomarker expression (Fig. 1D and E). Moreover, the expression of SARI impacted the in vitro cell motility significantly (Fig. 1F). Taken together, these data indicate that SARI is a potent EMT inhibitor.

### SARI Modulates the Glycogen Synthase Kinase (GSK)-3β-β-Catenin Signaling Pathway

**Figure 2:**
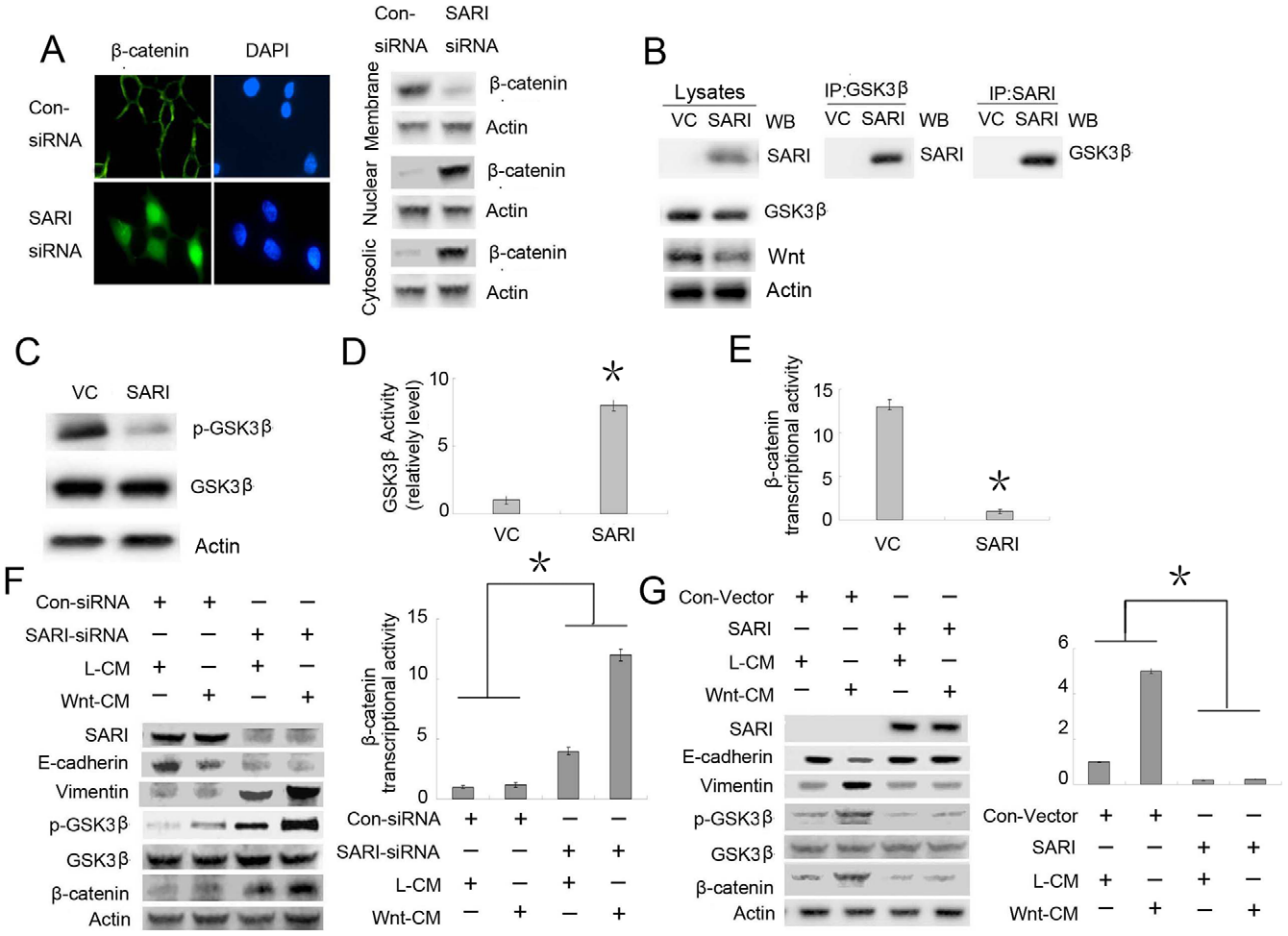
SARI activates the GSK-3β/β-catenin pathway and antagonizes Wnt-mediated EMT. (A) SARI prevents β-catenin nuclear translocation. NCI-H1650 cells were transfected with control or SARI-siRNA. The subcellular localization of β-catenin was visualized by confocal microscopy (magnification: 500×) and western blot. (B) SARI is associated with GSK3β. GLC-82 cells were transfected with the control vector (VC) or SARI. Cell lysates were immunoprecipitated with GSK-3β; then, SARI antibodies were probed with SARI or GSK3β antibodies. SARI can also inhibit Wnt protein expression (C) SARI inhibits GSK-3β phosphorylation at S9. GLC-82 cells were treated with the VC or SARI, and cell lysates were blotted with p-GSK-3β (S9) and GSK-3β antibodies. β-Actin was used as a loading control. (D) SARI activates GSK-3β kinase activity. Kinase activities were determined as described in Materials and Methods. Relative GSK-3β kinase activities were represented as the mean ± SEM from each sample after normalizing with untreated GLC-82 cells. Asterisks indicate statistically significant differences in GLC-82 cells versus the GLC-82 cells transfected with SARI cells (P<0.01). (E) SARI inhibits β-catenin/TCF transcriptional activity. GLC-82 cells were treated with VC and SARI and transfected with TCF-responsive promoter reporter (TOP-flash) or nonresponsive control reporter (FOP-flash); then, the luciferase activity was measured by the ratio of TOP and FOP. Relative luciferase activity is represented as the means ± SEM from each sample after normalizing with control ( = 1). The asterisk indicates statistically significant difference in GLC-82 cells versus the GLC-82 cells transfected with SARI cells (P<0.01). (F) Knockdown of SARI inactivates GSK-3β, promotes β-catenin activity, and enhances Wnt-induced EMT in NCI-H1650 cells. Cells were cotransfected with the control or SARI-siRNA and TOP or FOP and then treated with L- or Wnt-CM. Cell lysates were subjected to western blot. Relative luciferase analysis was performed as described above. Asterisks indicate statistically significant difference in cells transfected with control siRNA cell versus SARI-siRNA (P<0.05). (G) In contrast, restoring SARI expression in GLC-82 cells (SARI-negative cells) prevented Wnt-induced EMT. Asterisks indicate statistically significant differences in cells transfected with the control vector versus SARI.

To better understand the possible mechanism of SARI in EMT responses, we examined the effect of SARI on the GSK-3β-β-catenin signaling pathway. In canonical Wnt pathways, GSK-3β–mediated β-catenin degradation is inhibited, leading to an accumulation of β-catenin in the nucleus that further transactivates β-catenin/T-cell factor (TCF) target genes. Thus, the hallmark of β-catenin signaling in both normal and neoplastic tissues is nuclear translocation. By knocking down endogenous SARI levels with siRNA, we observed the accumulation of cytoplasmic β-catenin, nuclear translocation of β-catenin and reduced membrane-associated β-catenin (Fig. 2A).

Moreover, after GLC-82 was transfected with SARI, GSK-3β appeared to directly associate with SARI, based on immunoprecipitation assays (Fig. 2B). Because SARI is not a phosphatase, the mechanism of GSK-3β activation by SARI may be mediated by a separate phosphatase associated within this complex. Moreover, GSK-3β activity was significantly elevated based on Ser9 (S9, negative regulatory site) phosphorylation levels and β-catenin/TCF transcriptional activity (TOP/FOP) decreased (Fig. 2C, D, and E).

Consistently, knocking down endogenous SARI in NCI-H1650 cells by transient transfection of SARI-siRNA increased GSK-3β phosphorylation (S9) and β-catenin/TCF transcriptional activity, which were further potentiated by Wnt treatment (Fig. 2F). Thus, SARI modulates GSK-3β-β-catenin signaling through the activation of GSK-3β by reducing S9 phosphorylation.

Wnt signaling is a key inducer of EMT during embryonic development and cancer progression. We tested whether manipulating SARI levels in various cell lines could modulate Wnt-induced EMT. Whereas Wnt only slightly elicited EMT (Fig. 2F, lanes 1 and 2) in NCI-H1650 cells, its effect on EMT increased significantly after endogenous SARI was knocked down by SARI-siRNA (Fig. 2F, lanes 3 and 4). In contrast, restoring SARI expression in GLC-82 (SARI-negative cell) cells prevented Wnt-induced EMT (Fig. 2G), strongly suggesting that SARI is an antagonist of Wnt-mediated EMT.

We further investigated SARI's effect on snail, twist and the TGF pathway. GLC-82 cells were transfected with a control vector or SARI, and the expression of Snail, Slug, Twist and TGFb was then detected by western blot. We found that SARI does not affect these proteins, so in our present study, we conclude that SARI regulates EMT though the GSK3β pathway (Fig. S1).

### SARI Activates GSK-3β By Regulating PP2A

**Figure 3:**
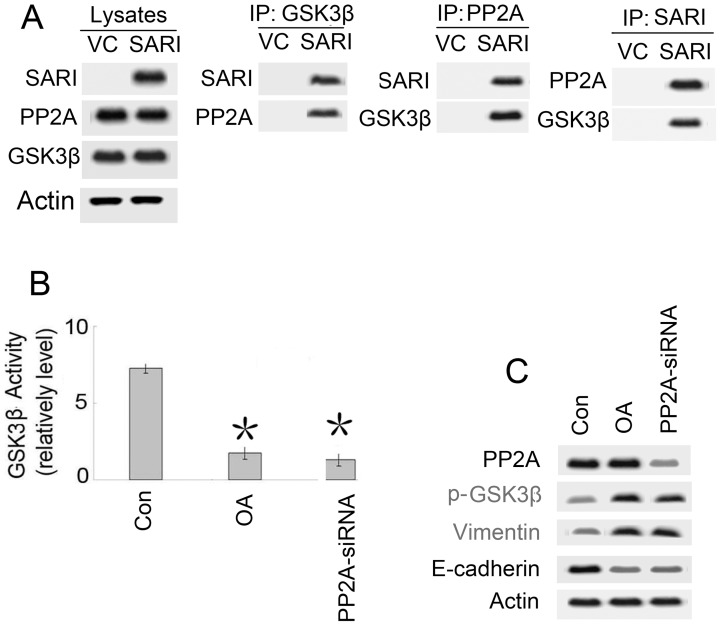
PP2A is critical for SARI-modulated GSK-3β-β-catenin signaling and EMT. (A) SARI complexes are associated with both GSK3β and PP2A. GLC-82 cells were transfected with control vector (VC) or SARI. Cell lysates were immunoprecipitated with GSK-3β, PP2A, and SARI antibodies and then probed with Flag and PP2A or GSK3β antibodies, respectively. (B) NCI-H1650 cells were cotransfected with PP2A-siRNA or treated with OA (okadaic acid) and then GSK-3β activity was determined. Both OA (okadaic acid) and PP2A-siRNA inactivate GSK-3β kinase activity (P<0.01). (C) The role of PP2A in SARI-modulated EMT and S9 phosphorylation of GSK-3β. GLC-82 cells transfected with either VC or DAB2IP were treated with OA (25 nM, 24 h) or cotransfected with PP2A-specific siRNA (100 pmol; 24 h). Cell lysates were subjected to Western blotting.

Based on co-immunoprecipitation (co-IP) (Fig. 2B), GSK-3β appears to directly associate with SARI. Because SARI is not a phosphatase, the mechanism of GSK-3β activation by SARI is likely mediated by a separate phosphatase associated with this complex. PP2A is a heterotrimeric complex containing a catalytic subunit, a structural subunit, and a variable regulatory subunit [18]. One study has shown that PP2A can regulate GSK-3β phosphorylation [19]. In our study, the co-IP data (Fig. 3A) indicated that SARI could form a complex with GSK-3β and PP2A.

To further assess the direct effect of PP2A on GSK-3β-β-catenin activity, we also examined the role of endogenous PP2A in SARI-modulated GSK-3β-β-catenin signaling. SARI-expressing cells were treated with the PP2A inhibitor, okadaic acid (OA), or PP2A-siRNA. Both the OA and PP2A-siRNA treatments abolished the SARI-mediated dephosphorylation of GSK-3β on S9 and regulation of EMT (Fig. 3C) and inactivated GSK-3β kinase activity (Fig. 3B). These data clearly indicate that PP2A is critical for SARI-mediated GSK-3β activation and MET responses. SARI inhibited GSK3beta activity though PP2A.

### β-Catenin Overexpression Reverses SARI-Mediated EMT

**Figure 4:**
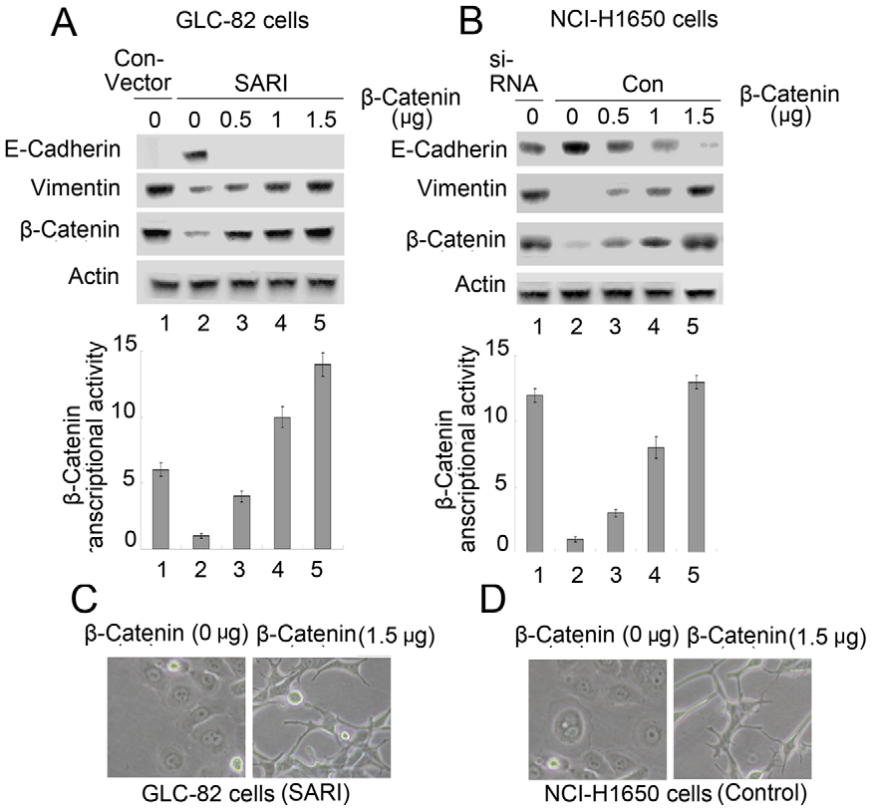
β-Catenin overexpression reverses SARI-mediated MET. Transfection of increasing amounts of β-catenin in GLC-82 (A) or NCI-H1650 (B) cells assayed at 24 h. Total cell lysates were probed with E-cadherin, vimentin, and β-catenin antibodies. β-Catenin/TCF transcriptional activity was assayed as previously described. (C) The morphology of GCL-82 cells as revealed by phase-contrast microscopy (magnification: 100×). (D) The morphology of NCI-H1650 cells was as revealed by phase-contrast microscopy (magnification: 100×).

Because SARI can activate GSK-3β and then lead to decreased cytosolic β-catenin protein levels and nuclear β-catenin transcriptional activity (Fig. 2A and E), we examined whether the inhibitory effect of SARI could be reversed by overexpressing β-catenin. In the SARI-transfected cells, increasing the dosage of β-catenin cDNA restored EMT as detected by the EMT markers and the increasing β-catenin transcriptional activity and morphology (Fig. 4A and C). Similarly, the elevated β-catenin protein levels and nuclear β-catenin transcriptional activity in NCI-H1650-KD cells also induced EMT in a dose-dependent manner (Fig. 4B). The morphology of these cells also changed (Fig. 4D).

### SARI Down-Regulation Promotes Tumor Progression and Metastasis

**Figure 5:**
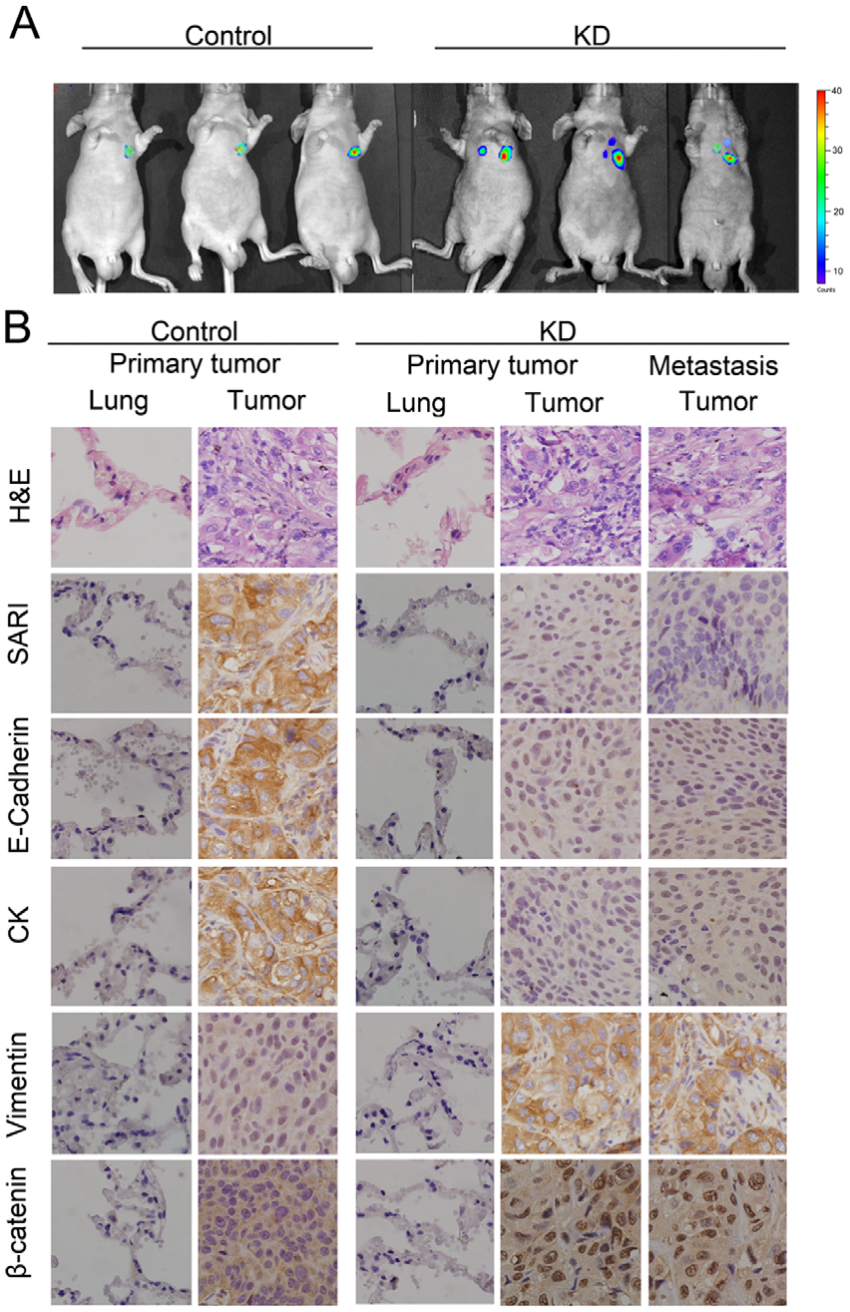
SARI down-regulation promotes tumor growth and metastasis. (A) Representative BLI imaging of mice bearing NCI-H1650-KD tumors with metastatic lesions. Mice (n = 5) were imaged 10 days later to determine local tumor growth and metastasis. (B) Representative H&E and IHC staining patterns. H&E staining showed primary tumor without detectable metastasis in control mice and lymph node metastases in mice bearing KD tumors 2 weeks post-injection (magnification: 100×). IHC showed the majority of the KD tumors with strong positive vimentin staining but weak E-cadherin, cytokeratin and SARI staining (magnification: 100×). IHC also showed that SARI prevents β-catenin nuclear translocation.

Because NCI-H1650 cells have low metastatic potential, and decreased SARI expression in these cells can initiate EMT (Fig. 1E and G), we examined the metastatic potential of KD- versus Con- expressing NCI-H1650 cells using an orthotopic mouse model. Stable luciferase activity was confirmed in each subline to ensure equal levels before injection. Bioluminescent imaging (BLI) was used to monitor tumor growth and the onset of metastases. One week after injection, BLI (Fig. 5A) clearly revealed multiple metastatic lesions at various sites in animals injected with NCI-H1650-KD cells. In contrast, control mice exhibited only small primary tumors 5 weeks post-injection, and none of the mice showed any signs of metastases (Fig. 5A). H and E data showed lung adenocarcinoma nude mice with or without metastasis (Fig. 5B). Immunohistochemistry (IHC) showed that the majority of tumor cells strongly expressed vimentin (Fig. 5B) but exhibited weak staining of E-cadherin and cytokeratin (Fig. 5B).

Moreover, there are differences in the sizes of primary tumors with and without SARI (Fig. S2A). These data provide strong evidence for the inhibitory role of SARI in lung adenocarcinoma metastases.

### SARI in primary adenocarcinoma lung tumors is correlated with a risk of lymph node metastasis

**Figure 6:**
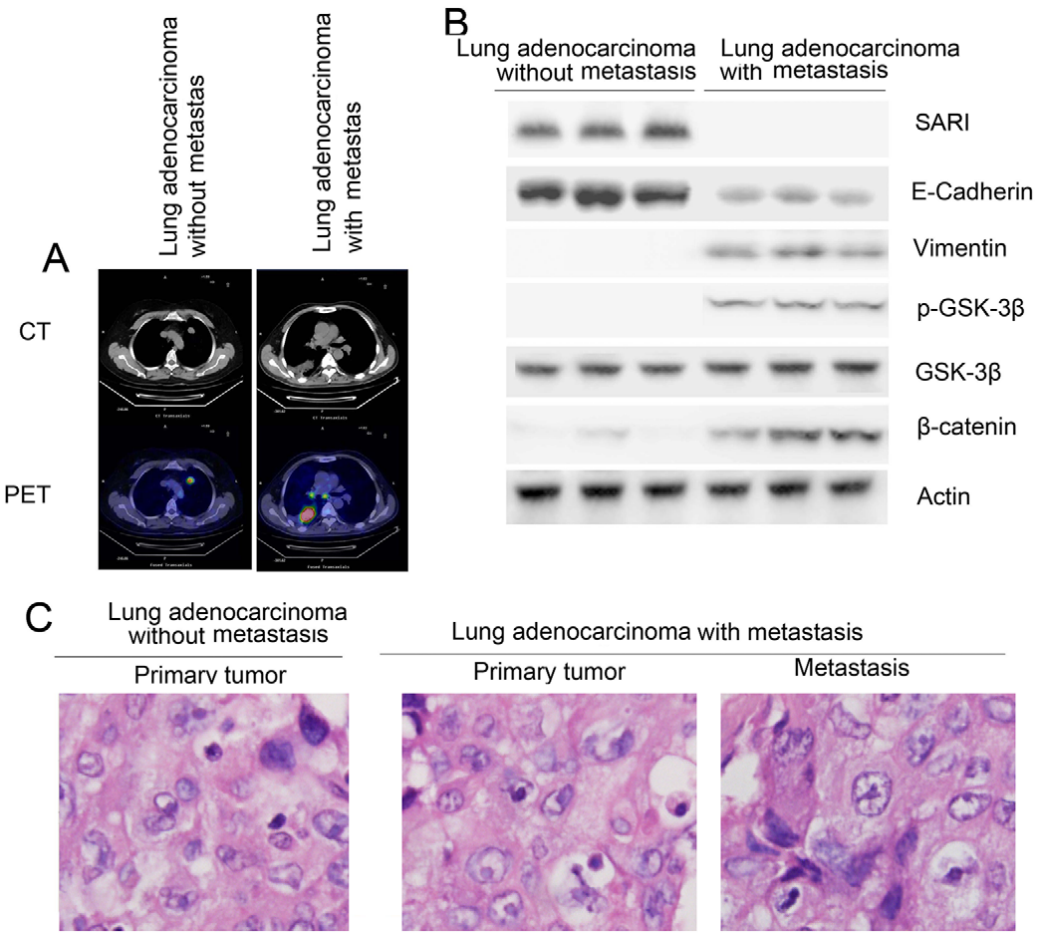
SARI regulates EMT in lung adenocarcinoma patients. (A) Different stage human lung adenocarcinoma specimens from lung adenocarcinoma patients with or without metastasis were chosen by PET/CT. (B) H&E data showing lung adenocarcinoma patients with or without lymph nodes metastasis. (C) Loss of SARI expression correlates with EMT marker changes in the clinical specimens from lung-cancer patients. Expression levels of SARI, E-cadherin, vimentin, and β-catenin protein as well as p-GSK-3β (S9) levels in normal (n = 10) and lung adenocarcinoma (n = 10) tissues were determined by western blotting. Densitometry was normalized with β-actin levels.

We further examined the relationship between SARI expression and EMT markers in lung adenocarcinoma patients. Different stages of human lung specimens from lung adenocarcinoma patients with or without lymph node metastases were selected by positron emission tomography-computed tomography (Fig. 6A). Based on the TNM Staging System for Lung Cancer, we selected six patients form stage I and seven patients from stage III; the detailed information for these patients is in Table 1. H&E data showed lung adenocarcinoma patients with or without lymph node metastasis (Fig. 6B). Loss of SARI and E-cadherin and increased vimentin, p-GSK-3β, and β-catenin levels were clearly detected in tissues from lung adenocarcinoma patients with lymph node metastasis (Fig. 6C). There was a significant correlation between the levels of SARI and E-cadherin (r = 0.8390) and an inverse correlation between the levels of SARI and vimentin (r = 0.7255) in all of the samples tested. Taken together, our human in vivo data are consistent with the in vitro data from various cancer-cell lines.

**Table 1:** Detailed patient information.

	sex	age	TNM	stage
Primary	Male	44	T_2_N_0_M_0_	I
Primary	Male	56	T_1_N_0_M_0_	I
Primary	Female	65	T_2_N_0_M_0_	I
Primary	Male	45	T_2_N_0_M_0_	I
Primary	Male	76	T_1_N_0_M_0_	I
Primary	Female	57	T_1_N_0_M_0_	I
Metastasis	Male	65	T_2_N_2_M_0_	III
Metastasis	Male	75	T_2_N_2_M_0_	III
Metastasis	Male	55	T_1_N_2_M_0_	III
Metastasis	Female	48	T_2_N_2_M_0_	III
Metastasis	Male	66	T_2_N_2_M_0_	III
Metastasis	Male	58	T_2_N_2_M_0_	III
Metastasis	Male	51	T_1_N_2_M_0_	III

“Primary” refers to patients without lymph-node metastasis, and “metastasis” refers to patients with lymph-node metastasis.

## Discussion

In this study, we showed that SARI functions as a critical protein in modulating GSK-3β-β-catenin signaling and EMT in human lung adenocarcinoma. The interaction of SARI with GSK-3β facilitates GSK-3β activation through S9 dephosphorylation. Activated GSK-3β decreases nuclear β-catenin accumulation and transcriptional activity, indicating the potent inhibitory function of SARI in Wnt-β-catenin signaling. Within the SARI-GSK-3β complex, SARI seems to be a negative regulator of Wnt-β-catenin signaling, which is the underlying mechanism for SARI function. The role of S9 phosphorylation of GSK-3β in Wnt/β-catenin signaling is still controversial. For example, the S9 phosphorylation of GSK-3β is not correlated with Wnt-mediated GSK-3β activity in certain cell types [20]. However, other studies have shown that many growth factors, such as insulin growth factor, transforming growth factor-β, and epidermal growth factor, can increase β-catenin accumulation through S9 phosphorylation of GSK-3β [21], [22]. The inactivation of GSK-3β through S9 phosphorylation is involved in hepatitis B virus-x protein (HBX)-mediated β-catenin stabilization in hepatocellular carcinoma cells [23]. In several tested lung adenocarcinoma cell lines, we showed that GSK-3β S9 phosphorylation was clearly involved in SARI-mediated β-catenin stability and transcriptional activity, suggesting that the effect of S9 phosphorylation on β-catenin signaling is cell-type dependent.

We further examined the relationship between SARI expression and EMT markers in lung adenocarcinoma patients. Loss of SARI and E-cadherin and increased vimentin and p-GSK-3β were clearly detected in tissues from lung adenocarcinoma patients who had lymph node metastasis (Fig. 6C). Moreover, SARI don't affected other EMT regulators including Snail, Slug, Twist and members of TGF pathway (Fig. S1).

In general, β-catenin has a dual role in EMT: it not only enhances cell–cell adhesion by associating with cadherin complexes in the adherens junctions of the cell membrane but also functions as a transcriptional coactivator aft.er interacting with TCF transcription factor complexes in the nucleus [2], [24], [25]. The induction of EMT by nuclear β-catenin has been explored during development in cell lines and tumors [2]. Several studies suggest that β-catenin–mediated transcription can induce *Slug* or *Twist1* gene expression [26], which further represses E-cadherin and thereby contributes to EMT. Our data show that loss of SARI in cells can lead to the accumulation of nuclear β-catenin (Fig. 2A and B). Thus, we believe that SARI can modulate the dynamic by switching between membrane- and nuclear-associated β-catenin (Fig. 2), which determines EMT [4]. Taken together, these data indicate that SARI is a key regulator in preventing EMT.

The majority of human carcinomas exhibit an epithelial phenotype. To break away from neighboring cells and invade adjacent tissue layers or peripheral lymph nodes, carcinoma cells often lose cell–cell adhesion and acquire motility. In general, 40% of the patients newly diagnosed with lung adenocarcinoma have local invasive cancer, and almost all of these patients eventually develop metastatic disease, accounting for most cancer deaths [1]. The detection of metastatic potential at an early stage should lead to an increase in disease-free survival rates. Regarding the clinical outcome of lung adenocarcinoma progression, the presence of lymph-node invasion has the lowest 10-year progression-free survival rate [27], [28], [29], [30]. Our orthotopic lung adenocarcinoma animal model (Fig. 5) demonstrates that mice bearing SARI-knockdown cells have a dramatic increase in the incidence of lymph-node metastases and the number of metastatic sites where tissues clearly exhibit mesenchymal characteristics. Wnt signaling has been identified as a determinant of lung adenocarcinoma metastasis to the brain and bones [31], [32], [33]. Similarly, our data indicate that the down-regulation of SARI can increase the propensity of lung adenocarcinoma cells to metastasize to lymph nodes (Fig. 5). Moreover, there are differences in the sizes of primary tumors with and without SARI, and there is also a difference in the proliferation of tumor cells with and without SARI, as SARI also plays a role in cell proliferation (Fig. S2).

In summary, this study delineates the functional role of SARI in EMT, which also explains how the loss of SARI in lung adenocarcinoma underlies the onset of aggressive metastatic lung adenocarcinoma. We believe that the assessment of SARI expression in lung adenocarcinoma specimens can be a valuable prognostic biomarker for the risk of lung adenocarcinoma metastasis and that the delineation of SARI function could provide a potential intervention strategy for lung adenocarcinoma metastasis.

## Materials and Methods

### Cell Culture and Clinical Specimens

The lung adenocarcinoma cell lines, including NCI-H1650, NCI-H1299, CRL-5908, NCI-H1975, CaLu-3, A549, GLC-82, PG49, and HTB-55, were obtained from ATCC. The cell lines were maintained in Dulbecco's Modified Eagle's Medium (DMEM; Invitrogen, USA) containing 10% fetal bovine serum (FBS; Invitrogen, USA).

The Institutional Review Board of China approved the retrieval of cancer specimens and the connection with the clinical data from our institute, approval ID 8435672. Cell lysates were subjected to western blot analysis or immunohistochemical staining.

### In Vitro Migration Assay

For the migration assays, 5×10^4^ cells were plated in the top chamber of a transwell (24-well insert; pore size  = 8 mm; Corning) and incubated with serum-free medium placed in the lower chamber. After incubation for 48 h, cells that did not migrate or invade through the pores were removed by a cotton swab, and cells on the lower surface of the membrane were stained with Cell Stain (Chemicon; Tokyo, Japan) and quantified by measuring the OD_560_.

### Analyses of the Wnt Signaling Pathway

WNT- and control-conditioned medium [Wnt-CM (ATCC number: CRL-2647) and L-CM] were collected according to the directions from ATCC and treated with cells for 24 h during the experiments. Cells were treated with Wnt-CM and L-CM (control) for 24 h, and the Wnt signaling activities were determined by performing various assays such as western blotting, a GSK-3β kinase assay (Boshida; Wuhan, China), a luciferase reporter gene assay (Chemicon; Tokyo, Japan), and fluorescence confocal microscopy (Sigma; BC, Germany).

### Orthotopic Animal Model and Imaging

All of the experimental procedures were approved by the Institutional Animal Care and Use Committee of China. The lungs of male nude mice (6–8 weeks of age) were exposed and injected with 5×10^5^ cells suspended in 20 μL of phosphate-buffered saline (PBS). One week after injection, the surgical staples were removed, and the tumor growth and local metastasis were monitored by bioluminescent imaging (BLI; Xenogen; CA, USA).

### Plasmid Constructs, Conditioned Medium, and Antibodies

Plasmids for SARI and PP2A were obtained from Sigma. For cDNA transfection, cells (5×10^5^ cells/well) were seeded in a 6-well plate (Costar) with 70–80% confluence before transfection. Transfection was carried out using Lipofectamine PLUS (Invitrogen, CA, USA) according to the manufacturer's instructions. WNT- and control-conditioned medium (Wnt-CM and L-CM) were collected according to the directions from ATCC and treated with cells for 24 h during the experiments. Anti-SARI polyclonal antibody was obtained from Biocompare. Okadaic acid (OA), Anti-GSK-3β, Anti-phospho-GSK-3β (S9), anti-actin, anti-E-cadherin, anti-β-catenin, and anti-vimentin were obtained from Sigma (BC, Germany). Anti-human specific pan-cytokeratin was purchased from Abcam (MA, USA). Anti-snail, anti-twist and anti-TGF were obtained from Invitrogen (CA, USA).

### siRNA Oligonucleotides and Delivery Methods

Three pairs of siRNA oligonucleotides for human SARI and PP2A were obtained from Invitrogen. siRNA oligonucleotides (20 μM) were transfected into cells using Lipofectamine 2000 (Invitrogen; CA, USA) according to the manufacturer's protocol.

### Immunoprecipitation and western Blot Analysis

For immunoprecipitation, transfected GLC-82 cells were washed twice with cold PBS and lysed in 1.5 mL of cold lysis buffer (50 mM Tris-HCl [pH 7.5], 150 mM NaCl, 0.1% Triton X-100, 1 mM sodium orthovanadate, 1 mM sodium fluoride, 1 mM sodium pyrophosphate, 10 mg/mL aprotinin, 10 mg/mL leupeptin, 2 mM phenylmethylsulfonyl fluoride, and 1 mM EDTA) for 20 min on ice. The immunocomplex was subjected to western blot analysis according to the manufacturer's protocol.

### GSK-3β Kinase Assay

A fluorescence peptide substrate-based assay was used to assess the GSK-3β kinase activity (Omnia Ser/Thr Recombinant Kit; Invitrogen; CA, USA). Briefly, the GSK-3β complex was prepared from the same amount of cell lysates by immunoprecipitation and then incubated with 10 μM of S/T Peptide substrate in kinase-reaction buffer (containing 1 mM ATP and 1 mM DTT) for 20 min at 30°C. Fluorescence intensity was recorded by measuring OD_485_ in a 96-well plate. Relative GSK-3β activity was calculated using untreated cells (equal to 1).

### Luciferase Reporter Gene Assay

For the reporter gene assay, cells seeded in 24-well plates were transfected with β-catenin, firefly luciferase reporter gene construct (TOP or FOP; 200 ng), and 1 ng of the pRL-SV40 renilla luciferase construct (as an internal control). Cell extracts were prepared 24 h after transfection, and the luciferase activity was measured using the Dual-Luciferase Reporter Assay System (Promega; Wisconsin, USA).

### Fluorescence Confocal Microscopy

NCI-H1650 cells were transfected with a control or SARI siRNA for 72 h. Cells were fixed in 4% formaldehyde and subjected to indirect immunofluorescence microscopy with anti-β-catenin. The fluorescein isothiocyanate (FITC)-conjugated anti-IgG was purchased from Molecular Probes. Confocal immunofluorescence microscopy (Olympus; Tokyo, Japan) was performed using an Olympus confocal microscope according to the manufacturer's protocol. The magnification used was 40×.

### BLI imaging

BLI was performed using the IVIS Imaging System (Xenogen; CA, USA). Images and bioluminescent signals were acquired and analyzed using the Living Image and Xenogen software. Briefly, in each imaging, 3 mice were anesthetized, injected with D-luciferin (150 mg/kg intraperitoneally), and then imaged 10 min after injection for 3 min.

### Histology and Immunohistochemical Staining

The tumors were removed, weighed, fixed in 5% formalin, and prepared for histological analysis. Consecutive tumor sections were stained with H&E, SARI, E-cadherin, vimentin, and β-catenin. Immunohistochemical staining was carried out using the ABC-staining Kit (Santa Cruz Biotechnology; CA, USA) and the secondary biotinylated antibody to mouse IgG (Invitrogen; CA, USA). Lung cancer patients' tissues were washed with PBS and then inflated and fixed with 10% buffered formalin. The sections were paraffin-embedded, sectioned in 5 μM, and stained with routine H&E.

### MTT Assay

For the MTT assays to measure the cell proliferation, NCI-H1650 cells (2×10^4^) were plated in 96-well plates (Boshida; Wuhan, China) and then transfected with control or SARI-siRNA. After incubation for 48 h, cells were stained with Cell Stain (Chemicon; Tokyo, Japan) and quantified by measuring the OD_560_.

### Statistical Analysis

The error bars in the graphical data represent the mean ± SEM. Student's two-tailed t test was used for the determination of statistical relevance between groups, and P<0.05 was considered significant.

## Supporting Information

Figure S1 GLC-82 cells were transfected with control vector or SARI. The expression of Snail, Slug, Twist and TGFb was detected by western blot.
(TIF)

Figure S2 SARI contributes to cell proliferation in vitro and in vivo experiment. Mice bearing NCI-H1650 or NCI-H1650-KD tumors were sacrificed, and the tumor size was measured. There are differences in the sizes of primary tumors with and without SARI (Fig, S2A). In NCI-H1650 cells transfected with control or SARI-siRNA, there is a difference in the proliferation of tumor cells with or without SARI (Fig, S2B).
(TIF)
