# Supplementary figures and images for: The Function of SARI in Modulating Epithelial-Mesenchymal Transition and Lung Adenocarcinoma Metastasis
Source: PLoS One. 2012 Sep 26;7(9):e38046. doi: 10.1371/journal.pone.0038046 (PMC3458851; doi:10.1371/journal.pone.0038046)

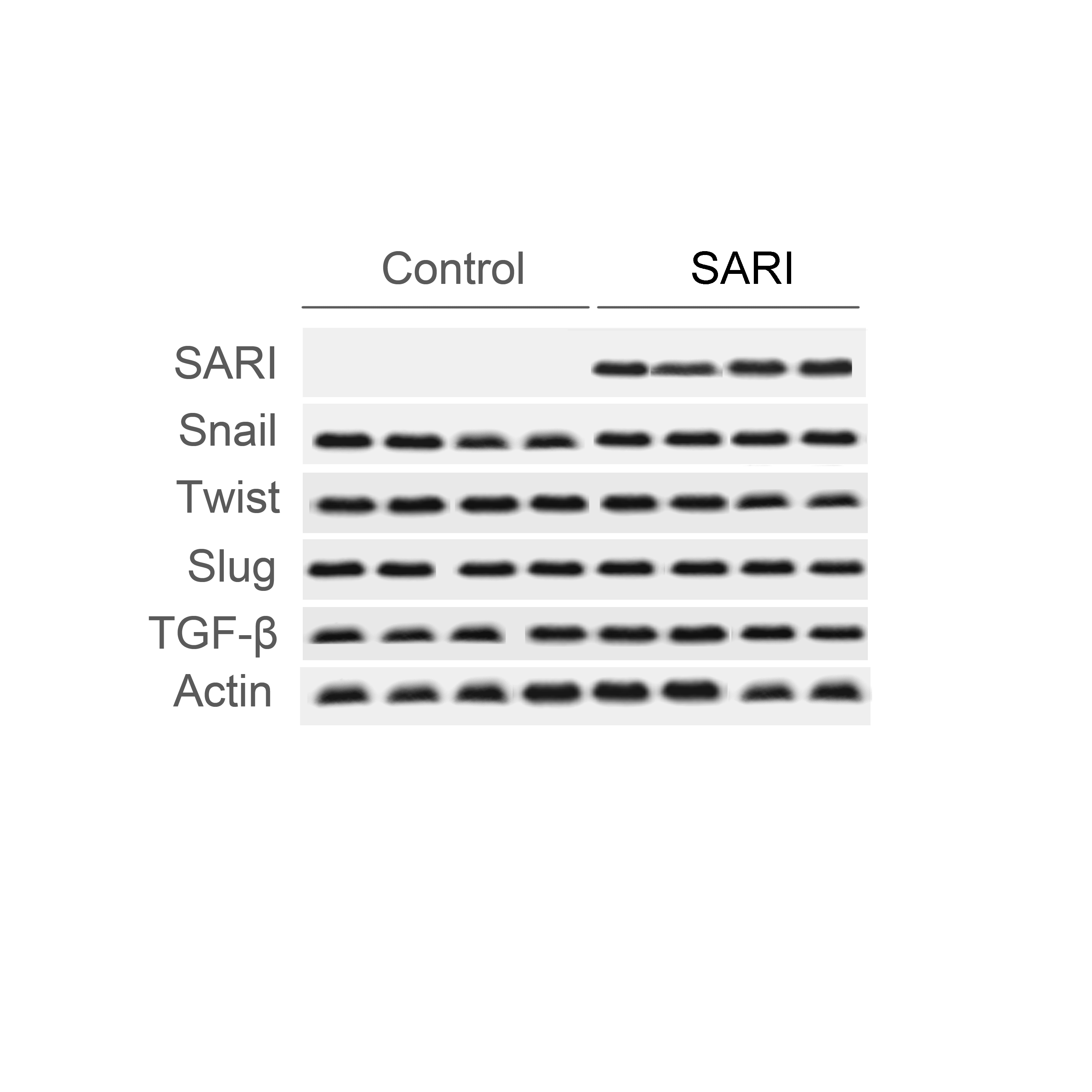

Supplement: Figure S1 — GLC-82 cells were transfected with control vector or SARI. The expression of Snail, Slug, Twist and TGFb was detected by western blot. (TIF) [file pone.0038046.s001.tif]

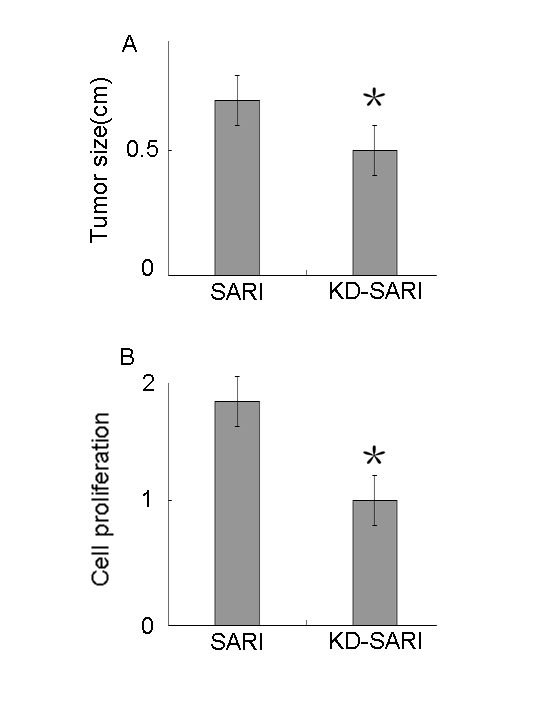

Supplement: Figure S2 — SARI contributes to cell proliferation in vitro and in vivo experiment. Mice bearing NCI-H1650 or NCI-H1650-KD tumors were sacrificed, and the tumor size was measured. There are differences in the sizes of primary tumors with and without SARI (Fig, S2A). In NCI-H1650 cells transfected with control or SARI-siRNA, there is a difference in the proliferation of tumor cells with or without SARI (Fig, S2B). (TIF) [file pone.0038046.s002.tif]
